# Strategies and solutions to address Digital Determinants of Health (DDOH) across underinvested communities

**DOI:** 10.1371/journal.pdig.0000314

**Published:** 2023-10-12

**Authors:** Casey Holmes Fee, Rachel Scarlett Hicklen, Sidney Jean, Nebal Abu Hussein, Lama Moukheiber, Michelle Foronda de Lota, Mira Moukheiber, Dana Moukheiber, Leo Anthony Celi, Irene Dankwa-Mullan

**Affiliations:** 1 Healthcare Consultant, Newton, Massachusetts, United States of America; 2 Research Medical Library, MD Anderson Cancer Center, Houston, Texas, United States of America; 3 Massachusetts Executive Office of Health and Human Services, Boston, Massachusetts, United States of America; 4 Simmons University, Boston, Massachusetts, United States of America; 5 Pulmonary, Critical Care and Sleep Medicine, Yale School of Medicine, New Haven, Connecticut, United States of America; 6 Department for BioMedical Research DBMR, Inselspital, Bern University Hospital, University of Bern, Bern, Switzerland; 7 Institute for Medical Engineering and Science, Massachusetts Institute of Technology, Cambridge, Massachusetts, United States of America; 8 University of Arizona, Tucson, Arizona, United States of America; 9 Picower Institute for Learning and Memory, Massachusetts Institute of Technology, Cambridge, Massachusetts, United States of America; 10 Division of Pulmonary, Critical Care and Pain Medicine, Beth Israel Deaconess Medical Center, Boston, Massachusetts, United States of America; 11 Department of Biostatistics, Harvard T.H. Chan School of Public Health, Boston, Massachusetts, United States of America; 12 Marti Health, Atlanta, Georgia, United States of America; 13 Department of Health Policy and Management, Milken Institute School of Public Health, The George Washington University, Washington, DC, United States of America; Kamuzu University of Health Sciences: University of Malawi College of Medicine, MALAWI

## Abstract

Healthcare has long struggled to improve services through technology without further widening health disparities. With the significant expansion of digital health, a group of healthcare professionals and scholars from across the globe are proposing the official usage of the term “Digital Determinants of Health” (DDOH) to explicitly call out the relationship between technology, healthcare, and equity. This is the final paper in a series published in *PLOS Digital Health* that seeks to understand and summarize current knowledge of the strategies and solutions that help to mitigate the negative effects of DDOH for underinvested communities. Through a search of English-language Medline, Scopus, and Google Scholar articles published since 2010, 345 articles were identified that discussed the application of digital health technology among underinvested communities. A group of 8 reviewers assessed 132 articles selected at random for the mention of solutions that minimize differences in DDOH. Solutions were then organized by categories of policy; design and development; implementation and adoption; and evaluation and ongoing monitoring. The data were then assessed by category and the findings summarized. The reviewers also looked for common themes across the solutions and evidence of effectiveness. From this limited scoping review, the authors found numerous solutions mentioned across the papers for addressing DDOH and many common themes emerged regardless of the specific community or digital health technology under review. There was notably less information on solutions regarding ongoing evaluation and monitoring which corresponded with a lack of research evidence regarding effectiveness. The findings directionally suggest that universal strategies and solutions can be developed to address DDOH independent of the specific community under focus. With the need for the further development of DDOH measures, we also provide a framework for DDOH assessment.

## Background

Digital health is an essential tool in the work to broaden and improve healthcare services across the globe, but technology is simultaneously capable of exacerbating inequities in society. This dueling relationship between digital health and equity is understood to be complex and nuanced, but still nascent as an independent field of study. To heighten awareness and solution-building around this topic, a global assemblage of healthcare professionals and scholars are proposing the use of a new term: Digital Determinants of Health (DDOH).

Digital Determinants of Health (DDOH) are the factors intrinsic to technology that when applied to the provision of healthcare services can have a major impact on health outcomes. The factors influencing DDOH include but are not limited to aspects such as ease of use, usefulness, interactivity, digital literacy, accessibility, affordability, algorithmic bias, technology personalization, data poverty, and information asymmetry [1].

While a formalized name is new, the connection between digital health and equity is a long-discussed phenomenon and over the last 2 decades has become increasingly recognized within major policy statements. These include the 2005 World Health Assembly where the World Health Organization (WHO) urged countries to draw up long-term strategic plans for incorporating digital health in a manner appropriate for each state’s health priorities and needs [2]. The Pan-American Health Organization defined digital inclusion as the “appropriate access, digital skills, and usability and navigability in the development of technological solutions” and proposed it as one of its 8 principles for the digital transformation of the health sector [3]. Most recently, WHO acknowledged the term “digital determinants of health” with examples of “literacy in information and communication technologies and access to equipment, broadband and the Internet” [4].

While acknowledged and highlighted broadly, digital health has mostly been studied as part of social determinants of health (SDOH). Independent exploration is burgeoning, but to date, there is no widely accepted or recognized definition of DDOH [5]. This gap in the field has grown increasingly worrisome when placed in the context of recent history. Specifically, that in 2020, digital health entered a new era with the mass adoption of telemedicine due to the Coronavirus Disease 2019 (COVID-19) pandemic. Further, applications for Artificial Intelligence (AI) and machine-based learning in healthcare are expanding rapidly [6–8]. Altogether, the healthcare industry is at an inflection point where the next decade of work will either improve equity across society through digital health or further worsen and cement the current divides.

To foster greater recognition of this issue, a group of healthcare professionals and scholars created a series of academic papers published in *PLOS Digital Health* that explores the definition, history, and current influences of DDOH in the industry. As part of that collaboration, we (the authors) created this final paper for the series on DDOH solutions.

## Methodology

### Objective

The objective of this paper is to gain a preliminary understanding of current academic knowledge of DDOH mitigation strategies and solutions, including their potential effectiveness. To meet this objective, we conducted a limited scoping review of recent academic literature related to digital health and underinvested communities.

### Scope

At the initiation of this project, we decided to keep the scope of the literature review as broad as practically possible. This choice meant no restrictions on the type of digital health technology discussed, location of the work, or specific disparity under review. Further, we included both conceptual and practiced ideas as well as patient facing technologies and those used by the healthcare providers and administrative staff. Finally, we wanted to look at solutions and strategies at all stages of the product life cycle from policy; design and development; implementation and adoption; and evaluation and ongoing monitoring.

### Search methods

A search of the literature was constructed by an accredited research librarian. Medline (Ovid), Embase (Ovid), Scopus, and Google Scholar were queried using natural language and controlled vocabulary terms for AI, telemedicine, digital health, digital literacy, computer proficiency, vulnerable populations, health outcomes, interventions, and mitigation techniques. We focused on highly cited references published since 2010 in English. The librarian then screened material for relevance and compiled a final list of 298 papers.

In parallel, the team was also reading and sharing articles on the subject found both in academic journals and trusted industry news outlets. Highly relevant articles were then included in the database. A total of 47 articles were identified in this manner, creating a final list of 345 papers.

### Literature review methods

Eight reviewers among this paper’s authors were identified to read and assess the articles. Each reviewer was given a preassigned set of papers selected at random from the initially compiled list. For each paper, the reviewer identified the author’s country based on university affiliation, type of study (conceptual guidance, literature review, etc.), type of digital health discussed (telehealth, digitalization, AI, etc.), and the type of underinvested community under focus in each article. The reviewer then chose to analyze the abstract or full article based on a combination of the relevance of the content and availability of the full text of the article. The reviewer documented any mentioned solutions in a commonly shared database hosted on Google Drive. Solutions were bucketed into one of 4 product life cycle stages for digital health technologies: policy; design and development; implementation and adoption; and evaluation and ongoing monitoring. The reviewer also documented any information on the effectiveness of the solution or strategy. No exclusion criteria were identified; therefore, all papers in the list were eligible to be reviewed.

### Analysis

Once the literature review was completed, the data were then aggregated by product life cycle stage. The information gathered on the solution effectiveness category was also aggregated. A single reviewer then analyzed the findings and summarized the DDOH strategies and the content on effectiveness.

### Key terms and definitions

Over the literature review process, we gained a better understanding of 2 key subjects that are worth greater explanation prior to reviewing the “Results” section of this paper: underinvested community categories and product life cycle stages.

I. Underinvested community categories

The term “underinvested community” refers to any group of people with a common trait that has historically received underinvestment in digital technologies that support and/or solve their healthcare needs. Over the scoping review, the reviewers created a list of underinvested community categories based on the topics covered in the reviewed papers. By the end of the assessment, we identified an extensive—though by no means exhaustive—list of 13 major groupings of underinvested community categories. These categories are described in Table 1.

**Table 1:** Underinvested community categories.

Underinvested Community Category	Definition	Common Usage in the Reviewed Papers	Example Paper
Age	Any age group or generation of patients or caregivers.	The research primarily focused on the elderly patient population. A few papers focused on pediatric concerns.	[9] Cresci MK, Jarosz PA. Bridging the Digital Divide for urban seniors: community partnership. Geriatr Nurs. 2010;31(6):455–463.
Culturally and Linguistically Diverse (CALD) background	Patients or caregivers who speak a different language or come from a different cultural background than the majority population in a given region or country.	The primary focus across the research reviewed was on patients and/or caregivers with limited English proficiency.	[10] Rodriguez JA, Casillas A, Cook BL, Marlin RP. The language of equity in digital health: Prioritizing the needs of limited english proficient communities in the patient portal 2.0. J Health Care Poor Underserved. 2021;32(2):211–219.
Urban/Rural	Patients living in an environment whose specific characteristics influence their health.	The papers primarily focused on patients living in rural environments with limited access to healthcare. Occasionally, health issues related to urban environments were mentioned.	[11] Cortelyou-Ward K, Atkins DN, Noblin A, Rotarius T, White P, Carey C. Navigating the Digital Divide: Barriers to Telehealth in Rural Areas. J Health Care Poor Underserved. 2020;31(4):1546–1556.
Low- and Middle-Income Countries (LMICs)	Patients and/or healthcare systems in countries with significant barriers to the delivery of healthcare services, including, but not limited to, digital infrastructure, literacy levels, and economic opportunity.	The papers covered the experiences of patients, providers, and caregivers in low- and middle-income countries (LMIC), primarily located in Central and South America, Asia, and Africa. The papers also covered organizations implementing digital health initiatives across LMICs.	[12] Stonbraker S, Haight E, Lopez A, Guijosa L, Davison E, Bushley D, et al. Digital Educational Support Groups Administered through WhatsApp Messenger Improve Health-Related Knowledge and Health Behaviors of New Adolescent Mothers in the Dominican Republic: A Multi-Method Study. Informatics. 2020;7(4).
Mental Health	Patients with mental or behavioral health concerns	The papers covered patient populations experiencing both mild and severe forms of mental health illness.	[13] Hoffman L, Wisniewski H, Hays R, Henson P, Vaidyam A, Hendel V, et al. Digital Opportunities for Outcomes in Recovery Services (DOORS): A Pragmatic Hands-On Group Approach Toward Increasing Digital Health and Smartphone Competencies, Autonomy, Relatedness, and Alliance for Those With Serious Mental Illness. J Psychiatr Pract. 2020;26(2):80–88.
Persons with Chronic Disease/Low Health	Persons with a major chronic illness. This category excludes persons with disabilities who are denoted in their own category.	The research focused on patient populations experiencing cancer, HIV, and stroke.	[14] Zhu C, Tran PM, Dreyer RP, Goldstein LB, Lichtman JH. Disparities in Internet Use among US Stroke Survivors: Implications for Telerehabilitation during COVID-19 and beyond. Stroke. 2022;29(2):E90-E91.
Persons with Disabilities	Persons with a physical or mental impairment that substantially limits one or more major life activities.	The papers covered persons experiencing significant disabilities such as hearing, sight, and mobility impairments.	[15] Valdez RS, Rogers CC, Claypool H, Trieshmann L, Frye O, Wellbeloved-Stone C, et al. Ensuring full participation of people with disabilities in an era of telehealth. J Am Med Inform Assoc. 2021;28(2):389–392.
Race/Ethnicity	Persons experiencing issues defined by their race and/or ethnicity	The papers covered general race/ethnicity/diversity concerns. Several papers focused on historically marginalized communities such as Black and Hispanic/Latino patients residing in the United States of America.	[16] Kim HS, Kim HJ, Juon HS. Racial/Ethnic Disparities in Patient-Provider Communication and the Role of E-Health Use. J Health Commun. 2021;26(3):194–203.
Social Determinants of Health (SDOH)	Persons with barriers to good health and/or high-quality healthcare services.	This topic covered any research that spoke to vulnerable populations more broadly covering high-level trends and guidance.	[17] Shah MK, Gibbs AC, Ali MK, Narayan KMV, Islam N. Overcoming the digital divide in the post-COVID-19 “reset”: Enhancing group virtual visits with community health workers. J Med Internet Res. 2021;23(7).
Sex/Gender	Persons experiencing issues defined by their sex and/or gender identity.	The research covered both women’s and men’s health topics.	[18] Figueroa CA, Luo T, Aguilera A, Lyles CR. The need for feminist intersectionality in digital health. Lancet Digit Health. 2021;3(8):e526-e533.
Sexuality	Persons experiencing issues defined by their sexual orientation.	The research primarily focused on patient populations of persons who have sex with same-sex partners.	[19] Hsiang E, Offer C, Prescott M, Rodriguez A, Behar E, Matheson T, et al. Bridging the Digital Divide Among Racial and Ethnic Minority Men Who Have Sex With Men to Reduce Substance Use and HIV Risk: Mixed Methods Feasibility Study. JMIR mHealth uHealth. 2020;8(4):e15282.
Socioeconomic Status	Persons experiencing issues affected by their access to social and financial resources.	This research focused on patients and caregivers living in low-income households.	[20] Sharma S, Barnett KG, Maypole J, Mishuris RG. Evaluation of mHealth Apps for Diverse, Low-Income Patient Populations: Framework Development and Application Study. JMIR Form Res. 2022;6(2).
Veterans	Former members of the military	The research focused on activities of the Veterans Association of the United States of America.	[21] Affairs OoPaI. VA expands Veteran access to telehealth with iPad services: VA; 2020 [updated September 15, 2020. Available from: https://www.va.gov/opa/pressrel/pressrelease.cfm?id=5521.

In the literature review, “not applicable” was used to denote papers that were not relevant to the topic and/or scope of the paper. “None” referred to papers that did not discuss any underinvested community. Finally, “Other” was used to capture papers that discussed an exceptionally unique or niche patient population.

Reviewers documented all underinvested community categories covered within a paper. For example, the paper “Bridging the Digital Divide for Urban Seniors: Community Partnership” [9] covered both the “Age” and “Geography” categories. The core measurement criteria are that the underinvested community category needed to be a primary focus of the paper. Minor references to other underinvested communities within the paper were not counted. Further, papers that more generally discussed “underinvested communities” or “disparities” broadly were measured under the “SDOH” category.

II. Product life cycle stages

While there are many and often unique phases for the development and implementation of any given digital health tool, we decided to consolidate the product life cycle into the 4 high-level buckets of policy; design and development; implementation and adoption; and evaluation and ongoing monitoring. These stages are described in Table 2. The logic was driven both as an economic choice to keep the review and data analysis feasible, but also by the sizable changes in key decision-makers associated with each phase. Further, we decided to include “policy” as a category that is atypical of product life cycle literature. We determined it was appropriate here due to the sizable influence that governments do and can have on the healthcare industry toward defining both healthcare services and equity/equality practices.

**Table 2:** Product life cycle stages.

Product Life Cycle Stages	Definition	Key Decision-Makers
Policy	The rules and/or public funds, which influence the development and implementation of digital health technology.	Policymakers Technology companies
Design and Development	The creation of hardware and software that supports or directly provides a healthcare service. This includes the conceptual design, data creation, workflow and usability studies, as well as feasibility testing.	Technology companies
Implementation and Adoption	The application of a digital health technology to patients or healthcare providers.	Healthcare organizations Technology companies Policymakers Patients/caregivers
Evaluation and Ongoing Monitoring	The evaluation measures and benchmarks to assess the social, ethical, economic and health impact, as well as effectiveness of a digital health technology.	All stakeholders as listed above

## Results

### Characteristics of articles identified and reviewed

A total of 345 articles were identified in the literature search and compiled in a random order. The first 175 articles were assigned to the 8 reviewers in amounts based on the reviewers’ offered capacity for assignments. Of the assigned articles, a total of 132 articles were reviewed by the end of the 2-month review period. A total of 213 articles were excluded from this limited scoping review.

Of the 132 papers reviewed, conceptual guidance papers were most common (36.1%) followed by research study (28.6%), real-world applications (20.3%), and literature reviews (15.0%). The underinvested community categories discussed across the papers were highly varied. Generalist papers (34.8%) spoke to social determinants of health broadly. A large collection of papers focused on the following underinvested community categories: chronic disease/low health (23.5%), socioeconomic status (23.5%), age (22.7%), urban/rural (21.2%), and culturally and linguistically diverse (CALD) backgrounds (19.7%). Less numerous underinvested community categories under focus across the papers included low- and middle-income countries (9.8%), sex/gender (7.6%), mental health (6.1%), persons with disabilities (5.3%), sexuality (2.3%), and veterans (2.3%). A limited number of papers had no applicable underinvested community category (7.6%). Due to the literature review being limited to English-language papers, most papers were written by authors with academic affiliations in North America, particularly from the United States of America (62.9%). The papers’ affiliations by continent were North American (73.5%), Asia (7.6%), Europe (7.6%), South American (2.3%), and Australia (1.5%). Six papers were identified as multicontinent or written by authors affiliated with universities located on different continents (4.5%). Across the papers, the reviewers conducted abstract-only reviews (23.4%), partial paper reviews (28.1%), and full paper reviews (48.4%) to find DDOH-related strategies and solutions.

In terms of digital health technologies explored, the largest portion of papers reviewed focused on telehealth/virtual care (38.3%). The second most frequent topic was digitalization (24.8%), which covered topics such as electronic health record adoption, computer/internet usage, or articles speaking to health IT more generically. The remaining papers’ health technology topics in order of frequency were health information exchange/portals (18.8%), AI/big data/clinical decision support (6.0%), and wearables/electronic patient-reported outcomes (ePRO)/immersive technologies (3.0%). A small number of articles were identified as other (3.8%) per covering highly niche topics such as attentional harms to mental health caused by persuasive technologies [22] or information needs of those with chronic illness from CALD backgrounds [23]. Another small batch of papers (3.0%) did not reference technology.

### Common DDOH strategies by product life cycle stage

#### Policy

Across the literature reviewed, many papers were written explicitly for policymakers or included policy recommendations within the discussion sections. It is clear that the policymaker and/or governing bodies are considered a key part in the work to drive (or enforce) inclusivity in the evolution of digital healthcare. The most common policy recommendations focused on accessibility, specifically the subcategory of affordability: how to pay for making digital healthcare accessible to underinvested communities. The other major factor discussed was the development of common standards with cyber security.

Payment recommendations focused on how governments should treat access to information technology and the internet as a human right per its increasing ties to public goods such as education and healthcare [24]. The implication is that governments need to provide, subsidize, and/or regulate pricing on the high-speed broadband internet access, hardware (i.e., computers, phones, etc.), and complementary supports (i.e., patient navigators, education, training, etc.) that help bridge the gaps for those with lower levels of digital literacy or with usability issues [25–27].

The policy guidance for equitable digital health access primarily focused on telemedicine—specifically for ensuring payment parity between in-person, video, and telephonic medical visits [10,11,26]. Studies found that underinvested populations were far more likely to use the telephone than video for visits during the height of the COVID-19 pandemic. If telephone visits regress to unpaid consultations, patient cohorts of those with socioeconomic and digital literacy barriers will receive lower access and quality of care than their peers [28,29].

Another payment solution promoted was the creation of reimbursements/billing codes for additional services that make telemedicine more inclusive [28]. These suggestions included the addition of interpreter services in a telehealth visit, clinical communications via the portal, email, or text, and time spent helping patients adapt to video-enabled telehealth.

Beyond payment, there’s a call for greater work around setting standards to ensure inclusivity in digital health [15,25,30]. A key area in this work is cyber security, particularly for any system that collects personal health information (PHI). Information security was discussed as an inclusivity need because security breaches hold higher negative consequences for patients with disabilities, behavioral health, chronic diseases, housing/food insecurity, etc. There is a call for further regulations, guidance, and standardization around cyber security to ensure that patients with more sensitive information can utilize digital health solutions with the same psychological safety as their peers [15].

#### Design and development

To enhance the inclusivity of digital health through design and development, many papers investigate or recommend specific features, technology, and devices that help to alleviate the digital gap for specific groups of people. For example, usage of Wearable Activity Trackers (WATs) to reduce the barrier of manual data input for older cancer survivors [31]. In reviewing the design suggestions, 3 key strategies emerged: (1) intentional design with and for users from historically underinvested communities; (2) management of user cost through usage of “low-tech” options; and (3) mindful construction of equitable databases and corresponding algorithms.

The most prominent strategy promoted across the articles is an upfront design decision to focus on and collaborate with users from historically underinvested communities. Technology companies are criticized for missing the mark on inclusivity in health tech as software designers are primarily not from the populations who most access or need healthcare services [32]. Across papers, there’s a call for more extensive collaboration with persons from populations who have historically received underinvestment to design and/or test digital health solutions. This strategy has many names such as participatory design, codesign, and user-centered design [18,33,34].

The goal of intentional design and collaboration is to create better-suited products for underinvested communities. Meeting this objective involves designing products to reduce overall physical and mental burden on the user through simplicity and automation of data wherever possible [15,32]. It can also mean use of personalization to enable patients to customize the functionality to a mode that is more accessible and/or adaptable to any physical, mental, or language need [25]. Finally, it can mean creation of team-based designs as persons with disabilities, limited English language proficiency, and other disparities often need caregivers and/or other care team members to participate closely in their care [15,25].

The second strategy—management of user cost—is an affordability goal. This strategy typically comes together in design choices that allow for the use of “low-tech” options. The most prominent example being the design choice to use text messages for healthcare communications as opposed to email and/or patient portals—which are known to have exceedingly low levels of uptake by underinvested communities [35,36]. Other examples include designing software and programs to work for simpler, lower-cost smart and/or cell phones, enabling functions that allow for apps to work in an offline mode, and settings that require devices to wait for access to Wi-Fi to avoid hefty broadband usage fees [37].

Finally, on the construct of data quality and information symmetry, there is an entire burgeoning scientific and highly technical field of study coming forward on equitable database creation and utilization of sensitive predictors such as race/ethnicity, socioeconomic status, and language [38]. Developers, particularly in AI/machine-based learning, should take into account the need for expertise and study in this field when creating digital health solutions. The goal is to create datasets that ensure accurate representation of populations as well as the appropriate use of sensitive constructs in algorithms. More insights on this rapidly evolving subject can be found in the corresponding paper in this DDOH Series “Bias in Artificial Intelligence Algorithms and Recommendations for Mitigation” by Lama Nazar and colleagues [39].

#### Implementation and adoption

There are numerous opportunities within the implementation of digital health solutions to minimize differences in DDOH across communities. While the literature on implementations was highly diverse from theoretical to real-world application, in aggregate numerous common strategies and themes did reveal themselves across the papers. For healthcare providers, there was guidance and stories that supported having stronger selection criteria when choosing products around usability for underinvested populations. Other recommendations focused on expanding access to solutions through complementary training and technology reimbursements. Finally, interpersonal elements such as cultural competence, communication, and trust-building were noted.

I. Selecting digital health for underinvested communities

At the point of the selection of a digital health solution, a key recommendation is to evaluate solutions for accessibility and usability for consumers with physical, financial, or other barriers to use. At the user level, this included both simplicity of software and hardware design as well as the ability to personalize features to incorporate physical, language, or other limitations. Other key usability factors included the ability to leverage low-tech devices, particularly text messaging for patient communication [35]. Finally, the literature recommended digital health solutions that support multimodel formats such as setups where patient reported health data can be reported via web, phone, or paper [40].

Finally, several papers considered the promise of AI, extended reality (XR), and machine-based learning toward building technologies that adjust and customize automatically to fit a patient’s needs overtime and with repeated use. For instance, for the elderly, there’s visionary statements of technology that can help provide custom prompts when the user is struggling and adjust programs based on monitored adherence to health behaviors. The hope is such technology will help to provide greater and better care at the home [32].

II. Expanding access

For digital health implementations, the main recommendation for operations was the creation of complementary training and support services to help patients, caregivers, and providers use the promoted digital health tool. Common strategies across training programs were reliance on culturally competent staff as well as the utilization of simulations and in-person trial runs with patients [41]. Particularly, simulations can be helpful for those with disabilities who need highly personalized configurations of digital health tools. Further, guidance emphasized the creation of programs specific to teaching digital literacy. For example, the Digital Opportunities for Outcomes in Recovery Services (DOORS) program provides hands-on training to persons with serious mental illness in order to allow them access to the virtual mental health services [13].

The other key operational decision in an implementation is considerations around accessibility. Similar to the policy recommendations, the literature spoke to the need for healthcare organizations to pay and/or provide the needed hardware, software, internet, etc. For example, the United States Veterans Association (VA) started the Connected Tablet program in 2016 providing cellular enabled iPads to qualifying veterans in order to support access to telehealth services [21]. Additional guidance supports incorporating access to technology as a critical need akin to providing parking vouchers to patients and families [36]. Utilization of partnerships across public, nonprofit, and for-profit industry were also encouraged toward how to expand access to the internet and computers for underinvested populations that could directly impact digital health services [9].

III. Building trust and relationships

Implementation of digital health technology is the critical phase in a product’s life cycle that provides the closest contact to the intended user. For this reason, interpersonal elements of trust and relationship building were a strong focus found across this scoping review.

Similar to the “Design” phase recommendation, many papers stressed the need to include representation from underinvested communities in the rollout of a digital health initiative. For example, through the creation of task forces or patient advocacy groups [36,42]. It also means relying heavily on culturally competent staff for providing training and ongoing support. Finally, a recommendation of training for all staff team members in cultural humility as we build up an expectation that time, training, and resources will be needed for any digital health initiative [23]. Expectation is that care team staff across all roles (medical to administrative) will need to gain skills in teaching and training a diverse set of patients and caregivers on digital health tools. Further, such training should be incorporated into medical and nursing schools as well as onboarding and continuing education across staff [43].

Building trust also was a theme that came out across papers in solution-building around digital health and disparities. For international entities looking to host solutions across countries, this included recommendations to have a deep understanding of a population’s trust in their country and healthcare system. Further not expecting a universal solution to low- and middle-income countries based on differing trust factors between individuals and their government and healthcare systems [44]. For individual care teams, there is a need to build trust and communicate trustworthiness, particularly when there’s a need for patients to share PHI electronically.

#### Evaluation and ongoing monitoring

For the design and deployment of digital health, ongoing measurement and monitoring will be needed to understand the impact on disparities in care. Reviewers collected the least content or information in this area compared to the other product life cycle stages. Most information around measurement was highly generalized, and only 2 specific evaluation tools were found both focused on software quality.

Of the papers reviewed, many referred to the need to prioritize the use of data for identifying disparities and tailoring improvement efforts [42]. This guidance included suggestions to conduct ongoing monitoring to ensure equity in use of digital health [25] or to evaluate whether disparities are being created, maintained, or worsened [45]. Others noted the need to engage directly with patients including screening patients for digital literacy and other barriers as well as conducting patient engagement scores and surveys. There also was a call to include health disparities as a key performance indicator on performance dashboards and related quality improvement interventions for digital health initiatives [27].

Only a few papers included specific suggestions on evaluation and performance measurement. For monitoring disparities, current guidance suggests utilization measurement of the digital health solution (internet, digital devices, portal use, etc.) with assessment for differences by sociodemographic and health characteristics [46]. For the field of telemedicine, the recommendation is to track which individuals appear to be absent from care, missing video visits, or relying on phone visits instead of video and develop outreach programs [27]. On patient assessment, measures around health literacy, digital literacy, language proficiency, and telemedicine access were suggested [36].

A handful of articles presented structured tools for the evaluation of the quality of digital health tools for underinvested communities. One such framework recently published is the evaluation of mHealth apps for diverse, low-income populations [20]. A second paper provided a framework for the evaluation of digital mental health solutions [47]. No tools were found that covered screening for barriers to digital health usage or ongoing measures to provide best practices for how to assess if disparities are narrowing or widening due to a digital health initiative.

Finally, there’s a call to ensure any measurement has an appropriate forum for reaction and response. For instance, as the data sets incorporating information from a wider cross section of the population grow, organizations will need to continuously refine their algorithms and retest. As patient data come in, the industry needs to ensure appropriate response loops to ongoing feedback and monitoring across all the product life cycle stages.

### Evidence of effectiveness of DDOH solutions

Regarding content related to DDOH solutions, the reviewers observed that the majority of papers primarily explored and/or researched the barriers to use of digital health platforms. A limited number of papers spoke to a real-world application of a solution or the use of a solution in the field. No papers were found that provided a case–control study of utilization of a digital health solution or cross-comparison of different solutions for a known digital disparity.

## Discussion

As a public good, the global healthcare industry is in the hot seat to ensure equity in the application of technology to its products and services. As the term Digital Determinants of Health (DDOH) becomes formalized, this paper is intended to serve as a check on what we currently know around strategies and solutions and where further study is needed. Key conclusions from this review are that a wide range of approaches to addressing disparities resulting from DDOH exist and that there’s directional evidence that universal approaches can be developed that can span across underinvested communities. At the same time, there is an outstanding need to develop ways to measure and monitor DDOH as well as conduct evaluations to determine the most effective solutions. Noting the significant gap in the area of measurement, we propose a starting framework for the assessment of DDOH in a person or community based on the themes that stood out the most in this limited scoping review.

### Breadth and depth of DDOH solutions

Given the intentionally broad scope of this paper, it came as no surprise that content on solutions and strategies was vast. While there were some highly generalizable papers that focused on SDOH factors and digital health broadly, most papers were highly targeted exploring a specific technology’s application to a defined community.

Based on the diversity and breadth of these papers, one of the greatest challenges to this scoping review was figuring out how to categorize the information by their commonalities. It was when we defined the underinvested community categories researched and segmented solutions by their product life cycle stage that the data could be summarized.

Looking forward, as DDOH evolves as a field, we will all benefit from exploring connections across the body of knowledge already compiled in this space. Through taking a more holistic view of the entire field, DDOH solutions and their translation to real-world application will likely happen much more quickly and effectively.

### Directional evidence supports common DDOH solutions

Given the wide variation in the research on DDOH, we approached this scoping review not knowing if the solutions would differ too greatly to draw any generalizable conclusions. We were pleased to discover that universal themes did emerge. For instance, access to the internet remained a common solution regardless if the paper was discussing how to provide every resident and business in New York City with broadband internet [48] or exploring last mile connectivity technologies for emerging markets [49]. With the caveat that this was a limited scoping review, current evidence directionally suggests that the healthcare industry does not need to approach mitigation to DDOH in silos. We likely can craft strategies, rules, guidelines, frameworks, and evaluations that scale across technologies and communities.

### Need for DDOH assessment and evaluation tools

Toward gauging which strategies and solutions will be the most effective depending on setting, we found a gap in the current research around measurement, assessment, and subsequent evaluation. In terms of content of the papers, a majority of research focused on measuring the barriers to adoption of digital health. Solutions to these barriers were then primarily discussed either theoretically or in unmeasured real-world application. Within the bounds of this limited scoping review, we did not find evidence of any assessment tools of DDOH or more structured research on DDOH solutions such as program evaluations, case–control studies, or cross-comparison of solutions. Consequently, while there are lots of solutions—mostly proposed—to making digital health more equitable, we currently do not know the extent to which different communities experience DDOH or which solutions will be most effective. This fosters our greatest concern that while there is great societal momentum right now to invest in health equity, we do not have the ability to optimize those funds nor measure success to support further investment.

That being said, one paper gave a hint that the right inclusivity effort can be incredibly effective. In a safety net hospital near New York City, a telemedicine initiative was set up to first engage patients to access a video visit through their patient portal. If the patient could not navigate through the portal, the practitioner would offer an “accessible option” to send the video connection link via text message. From November to December 2020, 10% of patients engaged in patient-portal video conferencing, while 56% utilized the text message link. The remaining 34% declined to participate in a video call and completed the visit by telephone instead [35]. Without the accessible text messaging solution, telemedicine via video would never have gotten off the ground at this hospital. Noting the small sample size and limited timeframe, findings like this give indication that the impact of accessible designs and implementations are potentially far bigger than what current business knowledge may suggest.

Funding needs to be directed toward DDOH evaluation research in order to understand the impact of different DDOH solutions. Such evidence is currently lacking and greatly needed in order to develop the best policies, solutions, and adoption programs for connecting underinvested communities with digital health opportunities.

### Proposed framework for DDOH assessment

To support the development of better DDOH assessment tools and metrics, we are proposing a 5-theme framework outlined in Table 3. These are based on the major themes discussed across the literature and could also be applied to the development of evaluation instrumentation as well.

**Table 3:** Proposed framework for DDOH assessment.

Theme	Definition	Questions
Accessibility	The understanding of a community or person’s ability to access—both physically and financially—the foundational technology needed for the practical use of digital health tools, including hardware, software, and the internet.	Does this community or person have access to all the foundational technology (internet, etc.) needed to use this tool?
Usability	The creation of software, hardware, and related services that account for the physical, mental, socioeconomic, racial, and language barriers of a community or person.	Is the tool designed and set up appropriately so that this community or person can feasibly use it?
Data and Algorithm Equity	The strength with which underlying data and algorithms accurately account for diverse and underinvested communities or persons.	Will this tool treat this community or person fairly? Is there any way in which the technology will act with bias toward this community or person?
Digital Literacy	The assessment of a community or person’s ability to effectively interact with digital technology to find, understand and apply information.	Does this community or person have the foundational computer skills to use this tool?
Trust	The knowledge of a community or person’s level of trust in the technology and/or the institutional parties offering/associated/affiliated with the digital health platform.	Does this community or person trust me? This technology? This institution?

### Connecting DDOH to health equity at large

In acknowledgment that even if major advancements are made in DDOH and more inclusive technologies are built and used, a person who is homeless or food insecure will still be left with significant barriers to health. DDOH is intentionally defined as a subset of SDOH per being a part of the solution for improving health across underinvested communities—but by no means a golden ticket. We believe there’s going to be tremendous value in studying the relationship between DDOH and SDOH and strongly recommend future efforts to better understand how DDOH ties into achieving health equity.

### Study strengths and limitations

The key strength of this study was the breadth of papers reviewed. By not limiting the scope based on disparity, geography, technology, study type, etc., we gained a broad set of perspectives on solutions for addressing DDOH. To our knowledge, this is a novel analysis within the field of digital health and equity studies. We are equally aware that we captured only a small portion of what is written—much less known—today on this subject matter.

In terms of this study, the largest limitation was the use of a limited scoping review methodology. This approach inherently meant that there was certainly missing relevant information and consequently large opportunity for potential bias in this paper. Even given these substantial limitations known at the outset, our team chose this methodology because it is the common approach for assessing large quantities of literature on an urgent issue in a low-resource setting. In short, a limited scoping review was the best fit for this kick-starter paper on DDOH solutions.

In regard to precedent, these types of expedited literature reviews are commonly found within academic literature and acknowledged to be cost-effective methods for knowledge discovery in time-sensitive/low-resource situations [50–53]. The research approach is called by many names including rapid review (RR); rapid evidence assessment; rapid systematic review, health technology assessment, rapid health technology assessment; miniature scoping review; brief scoping review; and limited review [52–56]. It refers specifically to “a form of knowledge synthesis in which components of the systematic review process are simplified or omitted to produce information in a timely manner” [52]. Modifications can include limiting the literature searched, limiting the inclusion criteria, and having only one person conduct parts of the research process [52]. While there is academic research available assessing the rapid review approach, there’s currently no universal methodology to rapid reviews in the same way there’s specific criteria for conducting a systematic review [51,56].

Such research is found in academic literature from as early as the 1960s, and with the pandemic, there’s been a dramatic rise in the number of rapid reviews in healthcare research [50–51]. Specifically, one study found that the number of publications in PubMed’s database (1960 to 2020) referring to “rapid review” nearly quadrupled in the single year between 2019 and 2020 (searched performed on March 16, 2021) [51].

Given the dual factors of the speedy advancement of healthcare technology combined with the dramatic rise in investments surrounding health equity, we felt there was sufficient urgency for this work to justify an expedited approach to the review.

Our other major modification was to term our approach a “limited scoping review” and not the commonly used term “rapid review.” Due to the novelty of the subject, a scoping review better described our objectives compared to the more honed focus of a systematic review (i.e., the method commonly associated with rapid reviews). While our approach still parallels a rapid review in many other regards, we decided to term our approach a “limited scoping review” to better communicate our method and intent for this paper.

Using a limited scoping review, the 3 specific limitations to our methods were as follows: (1) the review of a randomly selected set of 132 papers from the 345 identified; (2) allowing for reviewers to conduct a full, partial, or abstract-only read of the paper depending on relevance of the material; and (3) no cross-checks or reliability tests conducted to ensure consistency in the reviewer’s approach to the material. While we fully support the findings and conclusions outlined in this paper, these limitations should be accounted for, particularly the potential that there are great ideas for DDOH solutions not included in this paper, and there may be evaluations conducted on relevant solutions that were not identified. To this end, we hope this paper provides a foundation for larger scoping or systematic reviews of the literature to more extensively compile common approaches and existing evidence on DDOH evaluation.

## Conclusions

This series of papers on DDOH came together because of a recognized need to heighten acknowledgment of the interconnection between digital health and equity. In this paper, we described findings from a limited scoping review conducted to understand how to improve equity in the application of technology to healthcare services. We found that while there is a vast amount of research on the subject, the knowledge remains largely disconnected as the majority of work focuses on the specific technology and/or underinvested community. We believe there is great power in starting to consider the field of DDOH more holistically, particularly when it comes to solution building and the subsequent evaluation. Further, this study showed directional evidence that common strategies, frameworks, assessments, etc. can be made. Our hope is that this work helps inspire and provide the initial building blocks for others to start contemplating more universal approaches to addressing and measuring DDOH.

## Supporting information

S1 File Literature review—DDOH Strategies Data 2023.5.19: Dataset. (XLSX)
